# Thermally Driven Continuous Rolling of a Thick-Walled Cylindrical Rod

**DOI:** 10.3390/mi13112035

**Published:** 2022-11-21

**Authors:** Fayang Zhu, Changshen Du, Yuntong Dai, Kai Li

**Affiliations:** School of Civil Engineering, Anhui Jianzhu University, Hefei 230601, China

**Keywords:** thermally responsive, self-sustained, rolling, thick-walled cylindrical rod, energy conversion efficiency

## Abstract

Self-sustained motion can take advantage of direct energy extraction from a steady external environment to maintain its own motion, and has potential applications in energy harvesting, robotic motion, and transportation. Recent experiments have found that a thermally responsive rod can perform self-sustained rolling on a flat hot plate with an angular velocity determined by the competition between the thermal driving moment and the friction moment. A rod with a hollow cross section tends to greatly reduce the frictional resistance, while promising improvements in thermal conversion efficiency. In this paper, through deriving the equilibrium equations for steady-state self-sustained rolling of the thick-walled cylindrical rod, estimating the temperature field on the rod cross-section, and solving the analytical solution of the thermally induced driving moment, the dynamic behavior of the thermally driven self-sustained rolling of the thick-walled cylindrical rod is theoretically investigated. In addition, we investigate in detail the effects of radius ratio, heat transfer coefficient, heat flux, contact angle, thermal expansion coefficient, and sliding friction coefficient on the angular velocity of the self-sustained rolling of the thick-walled cylindrical rod to obtain the optimal ratio of internal and external radius. The results are instructive for the application of thick-walled cylindrical rods in the fields of waste heat harvesters and soft robotics.

## 1. Introduction

A self-sustained motion is a periodic motion continuously driven by a steady external stimulus, and has recently attracted widespread attention [1,2,3,4,5,6,7,8,9]. Research on self-sustained motion has potential applications in various areas, such as energy harvesting [10,11,12], subsidiary transport [13,14], self-cleaning systems [15], and small motors [16,17]. The application of this device can simplify equipment [18], optimize the control [19], and reduce the energy loss [20].

In recent years, based on various stimulus-responsive polymeric materials, including humidity-responsive polymeric materials [21,22,23], thermally responsive polymeric materials [24,25,26], light-responsive liquid crystal polymers [27,28,29], and chemically responsive hydrogels [30,31,32,33], rich motion modes have been proposed [34,35,36,37,38]. For instance, rolling [39,40], torsion [41,42], vibration [17,43], bending [44,45], and jumping [46,47,48,49,50] have been proposed according to the theory of self-sustained motion. The main challenges regarding the theoretical modeling of self-sustained motion are the interactions between different fields and the intrinsic nonlinearity of the system. Nevertheless, various theoretical models have been proposed for self-sustained motion to reveal the mechanism of self-sustained motion [6,51,52,53,54]. The main challenges regarding the theoretical modeling of self-sustained motion are the interactions between different fields and the intrinsic nonlinearity of the system. Nevertheless, various theoretical models have been proposed for self-sustained motion to reveal the mechanism of self-sustained motion [6,51,52,53,54].

Recently, the study of self-sustained motion for thermally responsive polymeric material has gained plenty of attention [4,17,55]. It has been observed experimentally that a polymer rod can roll or rotate steadily on a flat hot plate with uniform temperature. During its steady motion, the driving moment originates from the inhomogeneous expansion of the thermally responsive polymeric material. The self-sustained motion device can continuously obtain energy from the steady environment to maintain its motion, converting thermal energy into mechanical energy. Li et al. developed a thermodynamic model of a rod rolling on a hot plate and calculated the thermally induced deformation and stress fields of the rod during steady-state rolling, which also successfully predicted the bistability of the rod [55]. In addition, Du et al. developed a theoretical model of thermally driven self-sustained rotation of a hollow torus, and found that for a given heat flux, there exists an optimal radius ratio maximizing the energy efficiency [17]. Similar to other self-sustained motions [56,57,58], self-sustained rolling can harvest energy directly from the environment to maintain its own motion under the action of steady external stimuli [59,60,61], which has considerable application prospects in the field of soft robotics [13,15,62].

Similar to the overturning characteristics of the ring, the central part of the torus contributes less to the driving moment, but instead increases its friction moment and reduces the overturning angular velocity of the torus. Other things being equal, a rod with hollow section is expected to greatly reduce the friction moment and obtain a larger rolling angular velocity than a solid rod. It is possible for the rod to have an optimum radius ratio that maximizes the angular velocity and efficiency of the rod rolling, so it is of great interest to study thick cylindrical rod rolling on hot plate. In contrast to the hollow torus with constant curvature [17], the curvature of the steadily rolling thick-walled cylindrical rod varies with parameters such as heat flux, heat transfer coefficient, etc. Constrained by complex experimental conditions, this paper focuses on theoretical modelling to theoretically investigate the effects of various system parameters, including the radius ratio, on the angular velocity of a thermally responsive rod rolling itself on a flat plate; the effect of the radius ratio on the thermal conversion efficiency of a hollow self-sustaining rolling rod; and ultimately to predict the optimum internal and external radius ratio.

According to the existing literature, there are many studies on the self-rolling of the solid rod, but that of the thick-walled cylindrical rod has not been explored. The objective of this paper is to theoretically analyze the influence of a hollow section on a rod’s self-rolling, with the aim of playing a guiding role in improving the energy efficiency. The layout of this paper is as follows. In Section 2, the theoretical model of thermally driven self-sustained rolling of a thick-walled cylindrical rod on a hot plate is established, the temperature field on the rod cross-section is studied, and the analytical solution of the thermally induced driving moment of the rod is derived. In Section 3, the equilibrium equation for the steady-state self-sustained rolling of a thick-walled cylindrical rod is given. Meanwhile, the effects of radius ratio, heat transfer coefficient, heat flux, contact angle, thermal expansion coefficient, and sliding friction coefficient on the rolling angular velocity of the thick-walled cylindrical rod are investigated in detail. In addition, the effects of radius ratio and dimensionless heat flux on the energy efficiency are also investigated. In Section 4, a summary of this study is presented.

## 2. Thermally Induced Driving Moment of the Thick-Walled Cylindrical Rod

### 2.1. Temperature Field in the Steadily Rolling Rod

**Figure 1:**
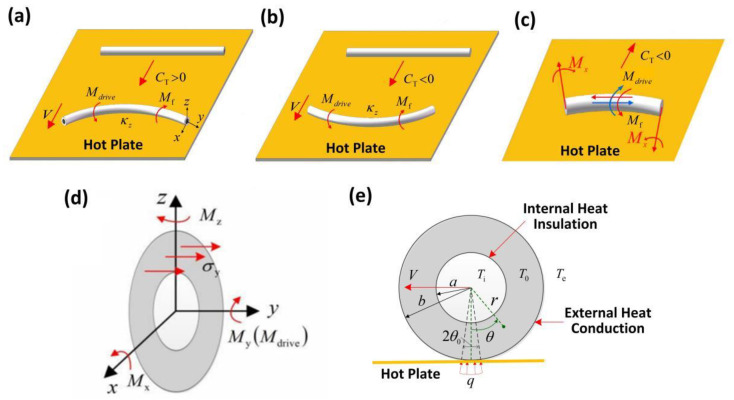
Schematic of a thermally driven rolling thick-walled cylindrical rod on a hot plate. (**a**) For thermal expansion materials, the thermal expansion coefficient $C_{T}$ is positive, and the rod rolls in a concave direction. (**b**) For thermal shrinkage materials, the thermal expansion coefficient $C_{T}$ is negative, and the rod rolls in a convex direction. (**c**) The driving moment applied to the thick-walled cylindrical rod and the friction moment acting on the rod. (**d**) The stresses on the cross-section of the thick-walled cylindrical rod and the moments in all directions. (**e**) The cross-section of the thick-walled cylindrical rod. Considering that the length of the rod is much greater than its thickness, we assume that the temperature distribution in the rod cross-section remains the same throughout the length of the rod. An inhomogeneous temperature field in the rod cross-section leads to uniform thermal expansion/shrinkage, which generates a driving moment $M_{\mathrm{drive}}$ that equilibrates with the friction moment $M_{f}$ and causes the thick-walled cylindrical rod to self-roll.

Our model is sketched in Figure 1. As shown in Figure 1a,b, a thick-walled cylindrical rod with internal radius a, external radius b, and length L was placed on a horizontal hot plate. For thermal expansion materials, the rod rolled in the concave direction (Figure 1a), and for thermal shrinkage materials, the rod rolled in the convex direction as shown in Figure 1b and Video S1. In Video S1, we use the nylon 6 material as an example. Figure 1c shows the driving moment applied to the thick-walled cylindrical rod and the friction moment acting on the rod. Figure 1d shows the stresses developed in the section of the thick-walled cylindrical rod and the moments in all directions. In Figure 1e, q indicates the heat flux, $T_{e}$ is the external environment temperature, and $\theta_{0}$ is the half contact angle.

In this study, our proposed model adopts the direct heating method. Of course, it is entirely possible to use indirect heating methods such as light or electromagnetic heating, as in the study of Ref. [14], the self-sustained rolling of solid LCE rod was achieved using light. Therefore, the light-powered self-sustained rolling of the hollow LCE rod will be a very worthwhile study. It is worth mentioning that, in order to simplify the problem, we assume that a thermal insulation layer is coated on the inner wall of the thick-walled cylindrical rod, and the inner wall of the thick-walled cylindrical rod was seen as an adiabatic boundary.

The coordinate systems in this paper are established with respect to plane, as shown in Figure 1. The Poisson effect of the thick-walled cylindrical rod and the rod end effect are neglected in the following analysis of this paper, so as to simplify the problem as a plane problem. Figure 1c shows the driving moment applied to the thick-walled cylindrical rod and the friction moment acting on the rod. In addition, since the deformation in this study is small, we assume that the contact angle is constant in the calculation. The rolling angular velocity of the thick-walled cylindrical rod at moment t is represented by ω(t). The heat transfer coefficient, the heat conduction coefficient, the mass density, and the specific heat of the rod are denoted by h, k, ρ and c, respectively. During the steady-state rolling of the thick-walled cylindrical rod, we have T(r,θ,t)=T(r,θ) and ω(t)=ω.

For the problem studied in this paper, we assume that except for the small area in contact with the hot plate, the remaining external surface of the thick-walled cylindrical rod is cooled by convection, while the internal surface of the rod is adiabatic. Due to the complexity of the thermal boundary of the rod, the thermal radiation effect has been neglected in order to simplify the analysis. To facilitate the calculation and analysis, we introduce the dimensionless parameters: radius ratio λ=a/b, $\bar{r}=r/b$, $\bar{T}=T/T_{e}$, $\bar{q}=qb/kT_{e}$, $\bar{h}=hb/k$, ${\bar{\beta }}_{m}=\beta_{m}b$, and $\bar{\omega }=\omega b^{2}/\psi$. Here, $\bar{T}$ is the dimensionless temperature filed, $\bar{q}$ is the dimensionless heat flux, $\bar{h}$ is the dimensionless heat transfer coefficient, ${\bar{\beta }}_{m}$ is the dimensionless air damping, and $\bar{\omega }$ is the dimensionless rolling angular velocity. In the steady-state rolling, the temperature field distribution in the rod cross-section can be expressed by following the previous work as [17,63,64]

(1) $$\begin{array}{l} \bar{T}\left(\bar{r},\theta \right)=(\pi \bar{h}+\bar{q}\theta_{0})\sum_{m=1}^{\infty }\frac{{\bar{R}}_{0}\left(\lambda ,\bar{h},\bar{r}\right)}{{\bar{F}}_{0}\left(\lambda ,\bar{h}\right)}\\ +2\bar{q}\sum_{n=1}^{\infty }\sum_{m=1}^{\infty }\frac{{\bar{R}}_{n}\left(\lambda ,\bar{h},\bar{r}\right)}{{\bar{F}}_{n}\left(\lambda ,\bar{h}\right)}\frac{sin\;n\theta_{0}}{n}\frac{cos\;n\theta +{\bar{\eta }}_{n}\left(\lambda ,\bar{h},\bar{\omega }\right)sin\;n\theta }{1+\mu_{n}^{2}\left(\lambda ,\bar{h},\bar{\omega }\right)} \end{array}$$

where ${\bar{R}}_{n}\left(\lambda ,\bar{h},\bar{r}\right)$, ${\bar{F}}_{n}\left(\lambda ,\bar{h}\right)$ and $\mu_{n}\left(\lambda ,\bar{h},\bar{\omega }\right)=\bar{\omega }n/{\bar{\beta }}_{m}^{2}\left(\lambda ,\bar{h}\right)$ are given by

(2) $${\bar{R}}_{n}\left(\lambda ,\bar{h},\bar{r}\right)={\bar{L}}_{n}J_{n}\left({\bar{\beta }}_{m}\bar{r}\right)-{\bar{V}}_{n}Y_{n}\left({\bar{\beta }}_{m}\bar{r}\right)$$

(3) $${\bar{F}}_{n}\left(\lambda ,\bar{h}\right)=B_{e}\left(\lambda ,\bar{h}\right)-\frac{B_{i}\left(\lambda ,\bar{h}\right){\bar{V}}_{n}^{2}}{{\bar{K}}_{n}^{2}}$$

where $B_{e}\left(\lambda ,\bar{h}\right)$ and $B_{i}\left(\lambda ,\bar{h}\right)$ are given by

(4) $${\bar{B}}_{i}\left(\lambda ,\bar{h}\right)={\bar{\beta }}_{m}^{2}\left(\lambda ,\bar{h}\right)-{\left(\frac{n}{\lambda }\right)}^{2}$$

(5) $${\bar{B}}_{e}\left(\lambda ,\bar{h}\right)={\bar{h}}^{2}+{\bar{\beta }}_{m}^{2}\left(\lambda ,\bar{h}\right)-n^{2}$$

with ${\bar{\beta }}_{m}\left(\lambda ,\bar{h}\right)$ being the positive root of the following characteristic equation [64]

(6) $${\bar{K}}_{n}{\bar{L}}_{n}-{\bar{V}}_{n}{\bar{W}}_{n}=0$$

in which ${\bar{K}}_{n}$, $\;{\bar{L}}_{n}$, $\;{\bar{V}}_{n}$ and ${\bar{W}}_{n}$ are [64]

(7) $${\bar{K}}_{n}=\frac{n}{\lambda }J_{n}\left({\bar{\beta }}_{m}\lambda \right)-{\bar{\beta }}_{m}J_{n+1}\left({\bar{\beta }}_{m}\lambda \right)$$

(8) $${\bar{L}}_{n}=\left(n+\bar{h}\right)Y_{n}\left({\bar{\beta }}_{m}\right)-{\bar{\beta }}_{m}Y_{n+1}\left({\bar{\beta }}_{m}\right)$$

(9) $${\bar{V}}_{n}=\left(n+\bar{h}\right)J_{n}\left({\bar{\beta }}_{m}\right)-{\bar{\beta }}_{m}J_{n+1}\left({\bar{\beta }}_{m}\right)$$

(10) $${\bar{W}}_{n}=\frac{n}{\lambda }Y_{n}\left({\bar{\beta }}_{m}\lambda \right)-{\bar{\beta }}_{m}Y_{n+1}\left({\bar{\beta }}_{m}\lambda \right)$$

with $J_{n}$ being the first-class Bessel functions, and $Y_{n}$ the second-class Bessel functions.

**Figure 2:**
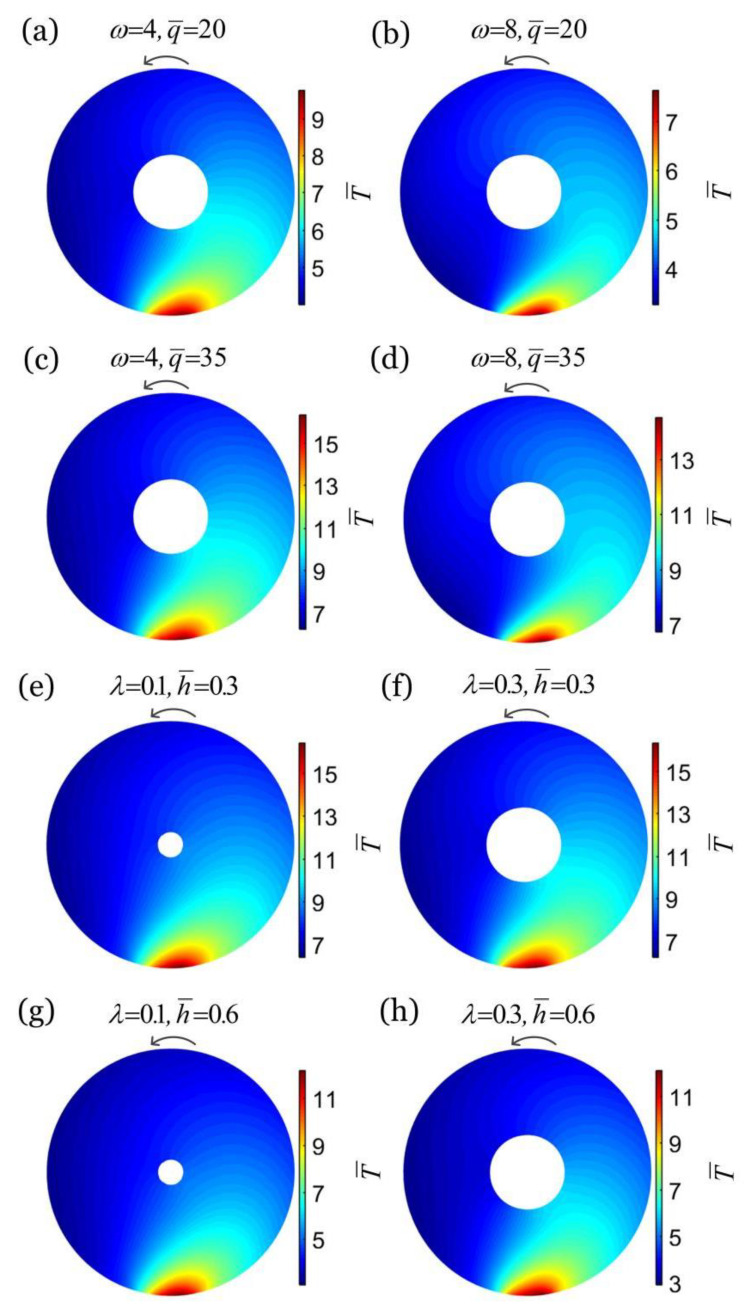
Temperature fields on the cross section of a thick-walled cylindrical rod in steady-state rolling for different combinations of rolling angular velocity $\bar{\omega }$ and heat flux $\bar{q}$: (**a**) $\bar{\omega }=4$, $\bar{q}=20$, (**b**) $\bar{\omega }=8$, $\;\bar{q}=20$, (**c**) $\bar{\omega }=4$, $\;\bar{q}=35$, (**d**) $\bar{\omega }=8$, $\;\bar{q}=35$, and different combinations of radius ratio λ and heat transfer coefficient $\bar{h}$: (**e**) λ=0.1, $\;\bar{h}=0.3$, (**f**) λ=0.3, $\;\bar{h}=0.3$, (**g**) λ=0.1, $\;\bar{h}=0.6$, (**h**) λ=0.3, $\;\bar{h}=0.6$. We set $\bar{h}=0.3$, λ=0.3, and $\theta_{0}=0.2$ in Figure 2a–d, and $\bar{\omega }=4$, $\bar{q}=35$ and $\theta_{0}=0.2$ in Figure 2e–h. In this case, the rolling velocity of the rod is one of the input parameters. When the thick-walled cylindrical rod rolls steadily, the non-uniformity of the temperature field on the cross section decreases with the increase in rolling angular velocity $\bar{\omega }$ and heat transfer coefficient $\bar{h}$, and increases with the increase in heat flux $\bar{q}$ and radius ratio λ.

From the accessible experiments [14,42], the typical values of material properties and geometrical parameters available for the experiments are presented in Table 1, and the estimates of dimensionless parameters are listed in Table 2. The deformation of the thick-walled cylindrical rod for the given parameters in this paper is small. Figure 2a–d shows the temperature fields on the cross section of a thick-walled cylindrical rod in steady-state rolling for different combinations of rolling angular velocity $\bar{\omega }$ and heat flux $\bar{q}$. We set $\bar{h}=0.3$, λ=0.3, and $\theta_{0}=0.2$. For a given heat flux $\bar{q}$, the temperature difference in the rod cross-section increases and then decreases with the increase in rolling angular velocity $\bar{\omega }$, and the temperature field tends to be uniform. In contrast, for a given rolling angular velocity $\bar{\omega }$, the temperature difference in the rod cross-section increases with the increase in heat flux $\bar{q}$, and the non-uniformity of the temperature field increases.

Figure 2e–h depicts the temperature fields on the cross section of a thick-walled cylindrical rod in steady-state rolling for different combinations of λ and $\bar{h}$. In the computation, we set $\bar{\omega }=4$, $\bar{q}=35$, and $\theta_{0}=0.2$. For a given heat transfer coefficient $\bar{h}$, along with the increase in radius ratio λ of the thick-walled cylindrical rod, we witness a slight increase in the temperature difference on the rod cross-section, and the temperature field tends to be inhomogeneous. This is because the smaller the radius ratio λ, the more unfavorable the heat transfer inside the thick-walled cylindrical rod, leading to a decrease in temperature in the low-temperature area and an increase in temperature in the high-temperature area. Additionally, for a given radius ratio λ, the temperature difference of the thick-walled cylindrical rod cross-section decreases with the increase in heat transfer coefficient $\bar{h}$, and the temperature field tends to be homogeneous. This is mainly owing to the fact that a larger heat transfer coefficient is conducive to heat transfer, resulting in a decrease in temperature in the high temperature region of the cross section and an increase in temperature in the low temperature region, so that the temperature field tends to be homogeneous.

### 2.2. Driving Moment for the Rolling of the Thick-Walled Cylindrical Rod

In this study, we assume that the thermally induced strain $\varepsilon_{T}$ is proportional to the temperature change on the rod cross-section for the purpose of analysis. The thermally induced strain is given by

(11) $$\varepsilon_{T}\left(r,\theta \right)=C_{T}[T\left(r,\theta \right)-T_{e}]$$

where $C_{T}$ is the thermal expansion coefficient of the material. For thermal expansion material, $C_{T}$ is positive, while for thermal shrinkage material, $C_{T}$ is negative.

Through applying the linear thermoelastic model, we can calculate the thermal stress σ(r,θ) on the rod cross-section along its normal direction as

(12) $$\sigma_{T}\left(r,\theta \right)=E\varepsilon_{T}\left(r,\theta \right)$$

where E is the elastic modulus of the material. The thermal bending moment of the rod around z-axis on the cross section can be described as

(13) $$M_{\mathrm{zT}}=\int_{a}^{b}\int_{0}^{2\pi }\sigma_{T}\left(r,\theta \right)rsin\;\theta rdrd\theta$$

Considering that the rod can bend freely in the lateral plane, the thermally driven lateral curvature can be calculated as

(14) $$\kappa_{z}=\frac{M_{\mathrm{zT}}\left(r,\theta \right)}{EI}$$

During the steady-state rolling of the thick-walled cylindrical rod, the total strain on the rod cross-section can be expressed as

(15) $$\varepsilon \left(r,\theta \right)=-\kappa_{z}rsin\;\theta$$

Thus, the axial stress on the rod cross-section can be obtained as,

(16) $$\sigma_{y}\left(r,\theta \right)=E\left[\varepsilon \left(r,\theta \right)-\varepsilon_{T}\left(r,\theta \right)\right]$$

and the direction of the axial stress on the cross-section of the rod is shown in Figure 1d.

The total bending moment of the rod around x-axis on the cross section can be described as

(17) $$M_{x}=\int_{a}^{b}\int_{0}^{2\pi }\sigma_{y}\left(r,\theta \right)rcos\;\theta rdrd\theta$$

Therefore, the total net moment applied on the thick-walled cylindrical rod can be expressed as [55]

(18) $$M_{\mathrm{drive}}=\kappa_{z}M_{x}L$$

For the convenience of computational analysis, we introduce ${\bar{C}}_{T}=C_{T}T_{e}$, $\bar{\sigma }=\sigma /E$, ${\bar{M}}_{x}=M_{x}/Eb^{3}$, ${\bar{M}}_{\mathrm{drive}}=M_{\mathrm{drive}}/Eb^{2}$, and ${\bar{\kappa }}_{z}=\kappa_{z}b$. By combining Equations (1) and (11)–(18), the driving moment of a thick-walled cylindrical rod in a steady rolling process can be rewritten as

(19) $${\bar{M}}_{\mathrm{drive}}=\frac{256\pi \bar{L}{\left(\bar{q}{\bar{C}}_{T}sin\;\theta_{0}\right)}^{2}}{\left(1-\lambda^{4}\right)}{\sum_{m=1}^{\infty }\eta_{1}\left[\frac{P\left(\lambda ,\bar{h}\right)}{\left(1+\eta_{1}^{2}\right){\bar{F}}_{1}\left(\lambda ,\bar{h}\right)}\right]}^{2}$$

The details of the derivation of Equation (19) are given in the Appendix A. In Equation (19), $P\left(\lambda ,\bar{h}\right)$ can be expressed as

(20) $$\begin{array}{cc} P\left(\lambda ,\bar{h}\right)= & \lambda^{2}J_{2}\left({\bar{\beta }}_{m}\lambda \right)Y_{2}\left({\bar{\beta }}_{m}\lambda \right)\\ & +J_{2}\left({\bar{\beta }}_{m}\right)\{\frac{{\bar{\beta }}_{m}}{2}[\mathrm{MeijerG}\left(\left\{\left\{-\frac{1}{2}\right\},\left\{-1\right\}\right\},\left\{\left\{-\frac{1}{2},\frac{1}{2}\right\},\left\{-\frac{3}{2},-1\right\}\right\},\frac{{\bar{\beta }}_{m}^{2}}{4}\right)\\ & -\lambda^{3}\mathrm{MeijerG}\left(\left\{\left\{-\frac{1}{2}\right\},\left\{-1\right\}\right\},\left\{\left\{-\frac{1}{2},\frac{1}{2}\right\},\left\{-\frac{3}{2},-1\right\}\right\},\frac{{\bar{\beta }}_{m}^{2}}{4}\right)]-Y_{2}\left({\bar{\beta }}_{m}\right)\} \end{array}$$

with $\mathrm{MeijerG}\left[\left\{\left\{s_{1}\ldots s_{n}\right\},\left\{s_{n+1}\ldots s_{p}\right\}\right\},\left\{\left\{t_{1}\ldots t_{m}\right\},\left\{s_{m+1}\ldots s_{q}\right\}\right\},z\right]$ being defined as the MeijerG function.

**Figure 3:**
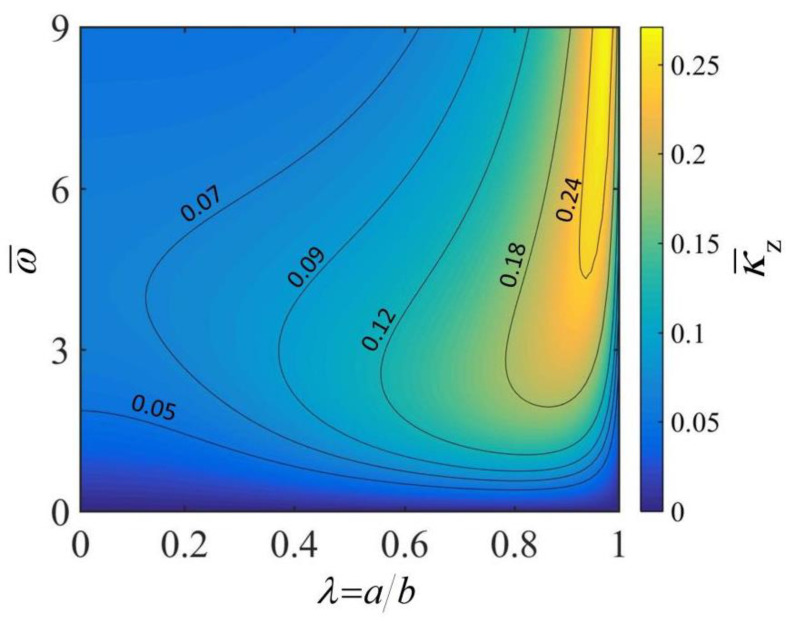
The effects of rolling angular velocity $\bar{\omega }$ and radius ratio λ of the thick-walled cylindrical rod on the lateral curvature ${\bar{\kappa }}_{z}$. In the computation, we set $\bar{h}=0.3$, $\bar{L}=10$, ${\bar{C}}_{T}=5\times 10^{-3}$, $\bar{q}=30$ and $\theta_{0}=0.2$. In this case, the rolling velocity of the rod is one of the output parameters. For a given angular velocity $\bar{\omega }$, there exists a radius ratio λ corresponding to an optimal lateral curvature ${\bar{\kappa }}_{z}$.

Figure 3 reflects the influence of $\bar{\omega }$ and λ of the thick-walled cylindrical rod on the lateral curvature ${\bar{\kappa }}_{z}$. In the calculation, we set $\bar{h}=0.3$, $\bar{L}=10$, ${\bar{C}}_{T}=5\times 10^{-3}$, $\bar{q}=30$ and $\theta_{0}=0.2$. It is apparent from the diagram that for a given radius ratio λ, the lateral curvature ${\bar{\kappa }}_{z}$ increases first and then decreases with the increasing rolling angular velocity $\bar{\omega }$. This is because the inhomogeneity of the steady-state temperature field in the rod cross-section increases and then decreases with the increase in rolling velocity (as shown in Figure 2). Meanwhile, the thermal bending moment and the thermally driven lateral curvature are positively correlated with the inhomogeneity of the temperature field (which can be derived from Equations (13) and (14)). For a given rolling angular velocity $\bar{\omega }$, in the process of enlarging the radius ratio λ, the lateral curvature ${\bar{\kappa }}_{z}$ shows a trend of rising first and then falling. This is because the inhomogeneity of the temperature field in the rod cross-section increases slightly with the increase in λ (as shown in Figure 2). It is easily concluded from the figure that under a given rolling angular velocity $\bar{\omega }$, there is an optimal radius ratio for the thick-walled cylindrical rod that maximizes the lateral curvature ${\bar{\kappa }}_{z}$. In addition, it is worth mentioning that the dimensionless heat transfer coefficient $\bar{h}$ can influence the lateral curvature ${\bar{\kappa }}_{z}$ of the thick-walled cylindrical rod by affecting the temperature field of the rod cross-section.

**Figure 4:**
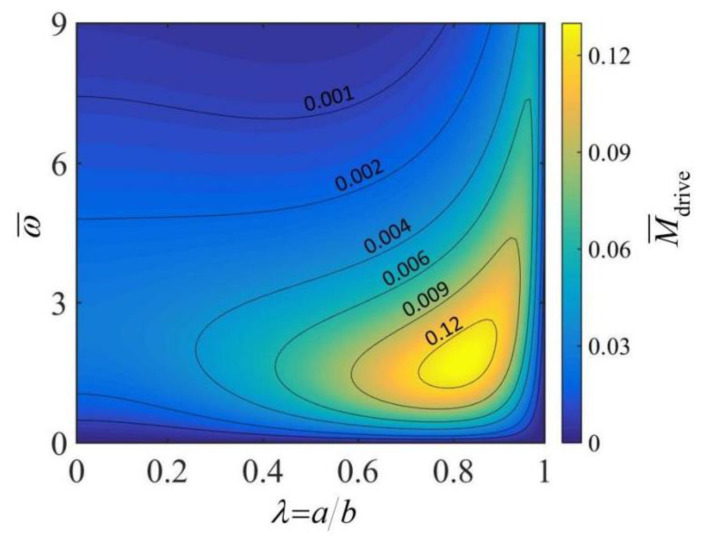
The effects of rolling angular velocity $\bar{\omega }$ and radius ratio of the thick-walled cylindrical rod λ on the driving moment ${\bar{M}}_{\mathrm{drive}}$. In the computation, we set: $\bar{h}=0.3$, $\bar{L}=10$, ${\bar{C}}_{T}=5\times 10^{-3}$, $\bar{q}=30$ and $\theta_{0}=0.2$. In this case, the rolling velocity of the rod is one of the output parameters. For a given angular velocity $\bar{\omega }$, there exists an optimal radius ratio λ corresponding to a maximum driving moment ${\bar{M}}_{\mathrm{drive}}$.

Figure 4 illustrates the effects of the rolling angular velocity $\bar{\omega }$ and the radius ratio λ of the thick-walled cylindrical rod on the driving moment ${\bar{M}}_{\mathrm{drive}}$. In the computation, we set: $\bar{h}=0.3$, $\bar{L}=10$, ${\bar{C}}_{T}=5\times 10^{-3}$, $\bar{q}=30$ and $\theta_{0}=0.2$. As can be seen from the figure, for a given radius ratio λ, the driving moment ${\bar{M}}_{\mathrm{drive}}$ increases and then decreases with the increase in rolling angular velocity $\bar{\omega }$. This is because the inhomogeneity of the temperature field of the rod cross-section increases and then decreases as the angular velocity increases, as shown in Figure 2, which ultimately leads to an increase and then a decrease in the driving moment ${\bar{M}}_{\mathrm{drive}}$. For a given rolling angular velocity $\bar{\omega }$, the driving moment ${\bar{M}}_{\mathrm{drive}}$ first increases and then decreases with the increase in radius ratio λ. This is because the temperature field in the rod cross-section becomes more nonuniform as the radius ratio λ grows larger. At a smaller radius ratio, the increased driving moment ${\bar{M}}_{\mathrm{drive}}$ due to the increase in temperature field inhomogeneity is large enough to compensate for the loss of driving moment ${\bar{M}}_{\mathrm{drive}}$ due to the reduction in the rod cross-section. For a given rolling angular velocity $\bar{\omega }$, there exists an optimal radius ratio of the thick-walled cylindrical rod corresponding to a maximum driving moment. However, we have to note that the actual maximum rolling angular velocity is dependent on the competition between the driving moment and the friction moment.

## 3. Self-Rolling of the Thick-Walled Cylindrical Rod on a Hot Plate

Considering the driving moment in Equation (19), the equilibrium equation during steady rolling of the thick-walled cylindrical rod is further derived in this section. Then, the effects of radius ratio, thermal expansion coefficient, heat flux, contact angle, heat transfer coefficient and sliding friction coefficient on the rolling angular velocity of the thick-walled cylindrical rod are studied in detail, and the critical value for triggering the self-sustained rolling is found. Furthermore, the dependence of the energy efficiency on the radius ratio is also investigated.

### 3.1. Equilibrium Equations

During the steady rolling, the surface of the thick-walled cylindrical rod is also subjected to sliding friction between the rod and the hot plate. The magnitude of the friction force on the thick-walled cylindrical rod can be expressed as

(21) $$F_{f}=C_{f}\rho g\pi \left(b^{2}-a^{2}\right)L$$

where $C_{f}$ denotes the sliding friction coefficient between the rod and the hot plate, and g is the gravitational acceleration. Moreover, the friction moment on the thick-walled cylindrical rod can be expressed as

(22) $$M_{f}=F_{f}b$$

During the steady-state rolling, the driving moment is equal to the friction moment. By combining Equations (19) and (22) and separating the fixed parameters, we can derive the equilibrium equation for the steady-state rolling as

(23) $$\frac{C_{f}\rho gb}{E{\left(\bar{q}{\bar{C}}_{T}sin\;\theta_{0}\right)}^{2}}=F\left(\lambda ,\bar{h},\bar{\omega }\right)$$

where

(24) $$F\left(\lambda ,\bar{h},\bar{\omega }\right)=\frac{256}{\left(1-\lambda^{2}\right)\left(1-\lambda^{4}\right)}{\sum_{m=1}^{\infty }\eta_{1}\left[\frac{P\left({\bar{\beta }}_{m}\right)}{\left(1+\eta_{1}^{2}\right){\bar{F}}_{1}\left({\bar{\beta }}_{m}\right)}\right]}^{2}$$

It can be observed from Equation (23) that although there are many parameters related to the angular velocity $\bar{\omega }$, including $C_{f}$, ρ, b, E, $\bar{q}$, ${\bar{C}}_{T}$, $\theta_{0}$, and $\bar{h}$, we only need to analyze the effects of three parameters, namely radius ratio λ, heat-transfer coefficient $\bar{h}$ and dimensionless parameter $\Omega =C_{f}\rho gr_{2}/E{\left(\bar{q}{\bar{C}}_{T}\sin\theta_{0}\right)}^{2}$, on the rolling angular velocity $\bar{\omega }$.

### 3.2. Angular Velocity of the Self-Rolling of Thick-Walled Cylindrical Rod

**Figure 5:**
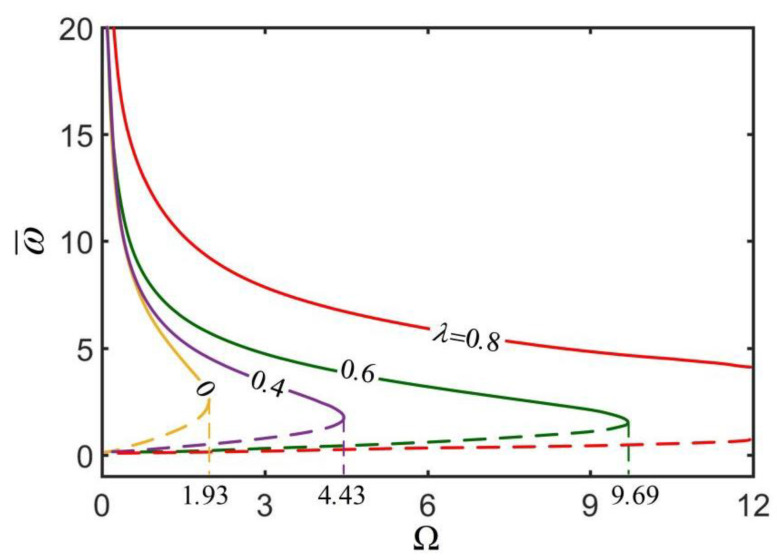
The effect of dimensionless parameter Ω on the rolling angular velocity $\bar{\omega }$ of the thick-walled cylindrical rod for $\bar{h}=0.3$. The rolling angular velocity $\bar{\omega }$ decreases with the increase in Ω. There is a critical $\Omega_{\mathrm{crit}}$ for maintaining the steady-state rolling of the thick-walled cylindrical rod, and $\Omega_{\mathrm{crit}}$ increases with the increase in radius ratio λ.

Figure 5 shows the effect of dimensionless parameter Ω on the rolling angular velocity $\bar{\omega }$ of the thick-walled cylindrical rod for different radius ratios λ. In the computation, we set $\bar{h}=0.3$. As can be seen from the diagram, there is a critical $\Omega_{\mathrm{crit}}$ for maintaining the steady-state rolling of the thick-walled cylindrical rod, and $\Omega_{\mathrm{crit}}$ increases with the increase in radius ratio λ. Obviously, the static state of the thick-walled cylindrical rod is always a steady state. When Ω increases to a critical value, the rod has another high-velocity steady state. Between static state and high velocity state, the rod has an intermediate velocity state, which is an unstable state because a perturbation of increasing (decreasing) the speed of the rod increases (decreases) its driving moment, leading to further increase or decrease in the rolling speed of the rod [55]. From Equations (21)–(23) and $\Omega =C_{f}\rho gr_{2}/E{\left(\bar{q}{\bar{C}}_{T}\sin\theta_{0}\right)}^{2}$, it can been seen that the dimensionless parameter Ω increases as the friction moment $M_{f}$ increases or as the driving moment $M_{\mathrm{drive}}$ decreases, so that the rolling angular velocity decreases.

Taking $\Omega =C_{f}\rho gr_{2}/E{\left(\bar{q}{\bar{C}}_{T}\sin\theta_{0}\right)}^{2}$ into consideration, we can also conclude that the rolling angular velocity $\bar{\omega }$ will increase with the increases in heat flux $\bar{q}$, contact angle $\theta_{0}$, thermal expansion coefficient ${\bar{C}}_{T}$, external radius b, and elastic modulus of the material E, and that conversely, it will decrease with increases in sliding friction coefficient $C_{f}$ and mass density ρ. The effects of these parameters on the rolling angular velocity of the thick-walled cylindrical rod are consistent with that of the solid rod on a hot plate [7,55].

**Figure 6:**
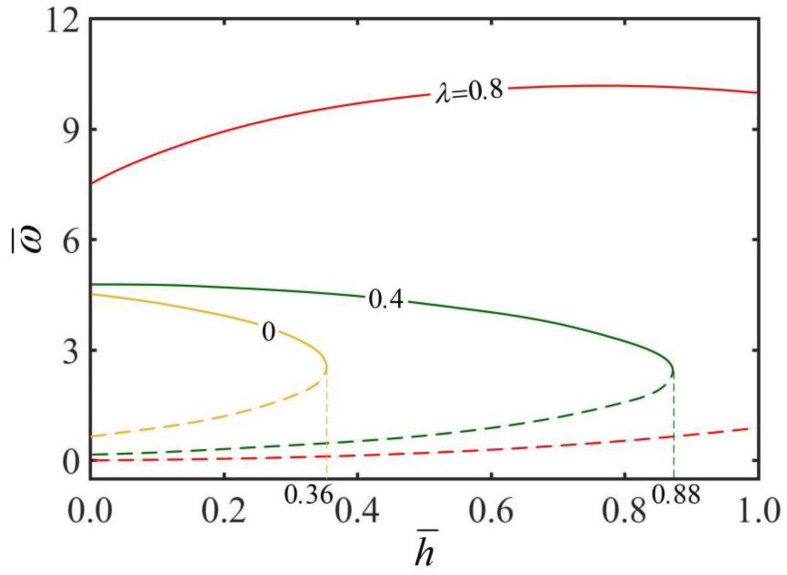
The effect of heat transfer coefficient $\bar{h}$ on the rolling angular velocity $\bar{\omega }$ of the thick-walled cylindrical rod for Ω=4. The rolling angular velocity $\bar{\omega }$ increases first and then decreases with the increase in heat transfer coefficient $\bar{h}$. There is a critical transfer coefficient ${\bar{h}}_{\mathrm{crit}}$ for keeping the steady-state rolling of the thick-walled cylindrical rod, and ${\bar{h}}_{\mathrm{crit}}$ increases with the increase in radius ratio λ.

Figure 6 presents the effect of heat transfer coefficient $\bar{h}$ on the rolling angular velocity $\bar{\omega }$ of the thick-walled cylindrical rod for different radius ratios λ. In the computation, we set Ω=4. As observed from the plots, there is a critical heat transfer coefficient ${\bar{h}}_{\mathrm{crit}}$ for keeping the steady rolling of the thick-walled cylindrical rod, and ${\bar{h}}_{\mathrm{crit}}$ increases with the increase in radius ratio λ. It is obvious that the static state of the thick-walled cylindrical rod is always a stable state. When the heat transfer coefficient $\bar{h}$ approaches the critical value, the rod presents another high-velocity steady state. Between the static state and high velocity, the rod has an intermediate rolling velocity, behaving as an unstable state. When the thick-walled cylindrical rod is rolling at a high velocity, under the given radius ratio λ, the increase in heat transfer coefficient $\bar{h}$ causes the rolling angular velocity $\bar{\omega }$ to increase first and then decrease. It is well understood from a physical point of view that for a smaller heat transfer coefficient $\bar{h}$, the inhomogeneity of the steady-state temperature field in the rod cross-section increases with the increase in heat transfer coefficient $\bar{h}$.

When the heat transfer coefficient $\bar{h}$ is large, the inhomogeneity of the steady-state temperature field decreases with the increase in heat transfer coefficient $\bar{h}$. For a given heat transfer coefficient $\bar{h}$, the rolling angular velocity $\bar{\omega }$ increases with the increase in radius ratio λ. This is because when the radius ratio λ increases, the heat transfer inside the thick-walled cylindrical rod is more favorable, and in turn the rolling angular velocity $\bar{\omega }$ is greater. For a larger heat transfer coefficient $\bar{h}$, the temperature difference on the rod cross-section decreases with the increase in heat transfer coefficient $\bar{h}$, and the temperature field tends to be homogeneous.

**Figure 7:**
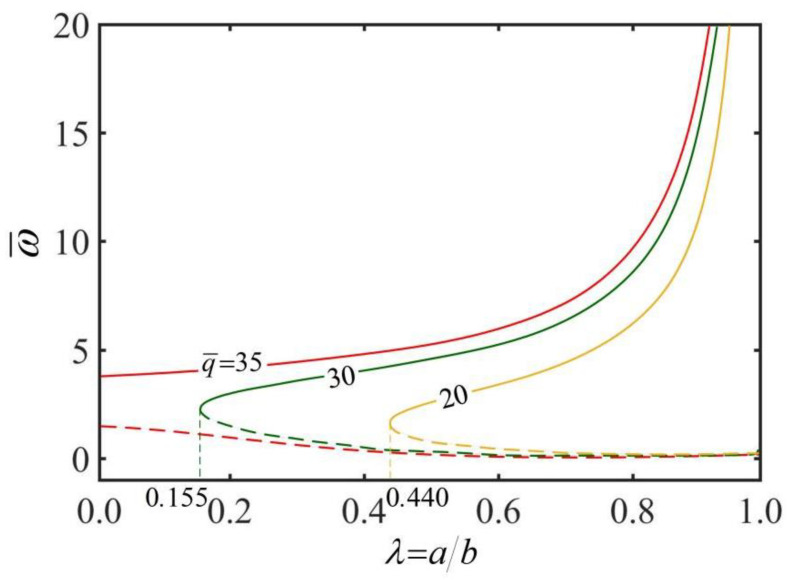
The effect of radius ratio λ on the rolling angular velocity $\bar{\omega }$ of the thick-walled cylindrical rod for $\bar{h}=0.3$. The rolling angular velocity $\bar{\omega }$ increases with the increase in radius ratio λ. There is a critical radius ratio $\lambda_{\mathrm{crit}}$ for keeping the steady rolling of the thick-walled cylindrical rod, and $\lambda_{\mathrm{crit}}$ decreases with the increase in heat flux $\bar{q}$.

Figure 7 shows the effect of radius ratios λ on the rolling angular velocity $\bar{\omega }$ of the thick-walled rod for different heat fluxes $\bar{q}$. In the computation, we set $\bar{h}=0.3$. As can be seen from the diagram, a critical radius ratio $\lambda_{\mathrm{crit}}$ exists to maintain the steady rolling of the thick-walled cylindrical rod, and $\lambda_{\mathrm{crit}}$ decreases as the heat flux $\bar{q}$ increases. It can be clearly seen that static state of the rod is always a stable state. When the radius ratio λ is increased towards a critical value, there is another stable state of the rod with high velocity. Between static and high velocity, there is an intermediate rolling velocity of the rod, which is an unstable state. For the thick-walled cylindrical rod rolling at high velocity, under a given heat flux $\bar{q}$, the rolling angular velocity $\bar{\omega }$ increases with the increase in radius ratios λ. This is due to the fact that the larger the radius ratio λ, the more conducive it is to the heat transfer inside the rod, the larger the rolling angular velocity $\bar{\omega }$.

For a given radius ratio λ, the rolling angular velocity $\bar{\omega }$ increases with the increase in heat flux $\bar{q}$. The reason for this phenomenon is that with the increase in heat flux $\bar{q}$, the temperature difference on the rod cross-section increases, and the non-uniformity of temperature field on the cross section increases, eventually resulting in the increases in driving moment ${\bar{M}}_{\mathrm{drive}}$ and rolling angular velocity $\bar{\omega }$.

### 3.3. Energy Efficiency of the Self-Rolling Thick-Walled Cylindrical Rod

The self-rolling system studied in this paper has the potential to be applied as a thermally driven motor or energy harvester. In the above theoretical model, the thick-walled cylindrical rod compensates the energy dissipated by damping through absorbing the thermal energy from the environment. If it is used as a thermally driven motor or energy harvester, we can regard the work performed by the damping force as the effective work output of the system. In the steady rolling of the thick-walled cylindrical rod, the input thermal power can be expressed in terms of heat flux density as $P_{\mathrm{in}}=2q\theta_{0}Lb$ and the effective power of the rod as $P_{e}=M_{f}\omega$. Combining Equations (21) and (22), the energy efficiency of a self-rolling thick-walled cylindrical rod is obtained from the following formula:

(25) $$\eta =\frac{C_{f}\rho g\pi \left(b^{2}-a^{2}\right)\omega }{2q\theta_{0}}$$

It can be clearly seen from Equation (25) that the energy efficiency η is related to many parameters, including $C_{f}$, ρ, b, a, q, and $\theta_{0}$. In the following, we take the combination of radius ratio λ and dimensionless heat flux $\bar{q}$ as an example to study the energy efficiency of the self-rolling thick-walled cylindrical rod. The typical values of parameters are $T_{e}=10^{\circ }C$, $h=0.3{W/m}^{2}/K$, $q=20{\sim 35\mathrm{kW}/m}^{2}$, k=0.05W/m/K, $b=10^{-3}m$, $\theta_{0}=0.2$, $\kappa =0.025m^{-1}$, $g=10{m/s}^{2}$, $\psi =1.2\times 10^{-6}m^{2}/s$, E=5MPa, $C_{T}=5\times 10^{-4}{/}^{\circ }C$, $\rho =1.3\times 10^{3}{\mathrm{kg}/m}^{3}$, and $C_{f}=0.45$.

**Figure 8:**
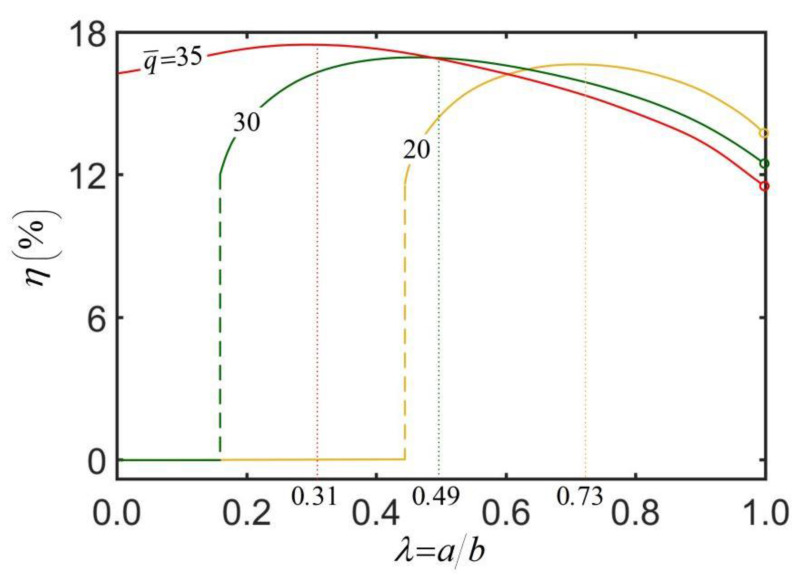
The dependence of energy efficiency η on the radius ratio λ and dimensionless heat flux $\bar{q}$. The parameters are $\bar{h}=0.3$, ${\bar{\kappa }}_{z}=0.025$, ${\bar{C}}_{T}=5\times 10^{-3}$, $C_{f}=0.45$, and $\theta_{0}=0.2$. There exists an optimal radius ratio that maximizes the energy efficiency of the thick-walled cylindrical rod, and the optimal radius ratio decreases as the heat flux increases.

Figure 8 illustrates the dependence of energy efficiency η on the radius ratio λ and dimensionless heat flux $\bar{q}$. The parameters are set: $\bar{h}=0.3$, ${\bar{\kappa }}_{z}=0.025$, ${\bar{C}}_{T}=5\times 10^{-3}$, $C_{f}=0.45$ and $\theta_{0}=0.2$. The results show that for a stationary thick-walled cylindrical rod, the energy efficiency of the rod is zero. When the thick-walled cylindrical rod undergoes a steady-state rolling, for a given heat flux $\bar{q}$, the energy efficiency increases first and then gradually decreases along with the increase in radius ratio. There exists an optimal radius ratio maximizing the energy efficiency of the rod, and the optimal radius ratio presents a certain decrease as the heat flux increases. This results from the competition between the kinetic energy and the heat dissipation, which both increase with the increase in the radius ratio. Therefore, the radius ratio of the thick-walled cylindrical rod is not as large as possible. In practical applications, it is necessary to seek a balance between the rolling angular velocity and the energy efficiency.

Table 3 lists the optimal radius ratio, the maximum energy efficiency of the thick-walled cylindrical rod, the energy efficiency of the solid rod and the energy efficiency improvement of the thick-walled cylindrical rod for three different heat fluxes $\bar{q}$ in Figure 8. As shown in Table 3, $\bar{q}=30$ corresponds to the maximum energy efficiency improvement, i.e., the increase from 0% to 16.95%. Overall, the results of this study show that, in practical applications, we can improve energy efficiency by adjusting the radius ratio of thick-walled cylindrical rod.

## 4. Conclusions

The study of self-sustained motion based on thermally responsive polymer materials has potential applications in soft robot, energy harvesting, etc. In this paper, by establishing a theoretical model of thermally driven self-sustained rolling of the thick-walled cylindrical rod, the effects of radius ratio, heat transfer coefficient, heat flux, contact angle, thermal expansion coefficient, and sliding friction coefficient on the rolling angular velocity of the thick-walled cylindrical rod were studied in detail. The main conclusions of this paper are now summarized as follows: (1) Similarly to the solid rod self-rolling on a hot surface, the thick-walled cylindrical rod has two stable states of static state and high-velocity rolling state, and one unstable state at intermediate rolling velocity. When the thick-walled cylindrical rod is in the high-velocity rolling state, the rolling velocity of the rod increases with the increase in radius ratio, and shows the same increasing trend with the increase in heat flux. (2) Moreover, the rolling velocity first increases and then decreases with the increase in heat transfer coefficient. (3) Especially, there exists an optimal radius ratio that maximizes the thermal conversion efficiency for a given heat flux. To summarize, the results of this paper are instructive for the application of thick-walled cylindrical rod in the fields of robot mobility and waste heat harvester.

## Figures and Tables

**Table 1:** Material properties and geometric parameters.

Parameter	Definition	Value	Unit
*T*_e_	External environment temperature	10	${}^{\circ }C$
a	Internal radius	${0\sim 10}^{-3}$	m
b	External radius	$10^{-3}$	m
h	Heat transfer coefficient	5~10	$W/m^{2}/{}^{\circ }C$
ψ	Thermal diffusion coefficient	$1.2\times 10^{-6}$	$m^{2}/s$
*k*	Heat conduction coefficient	0.05~0.1	$W/m/{}^{\circ }C$
*q*	Heat flux	$0\sim 35\times 10^{3}$	$W/m^{2}$
*ω*	Rolling angular velocity	0~4π	$s^{-1}$
L	Rod length	0.2	m
$C_{T}$	Thermal expansion coefficient	$5\times 10^{-4}$	$1/{}^{\circ }C$
E	Elastic modulus of the material	5	MPa
ρ	Mass density	$1.3\times 10^{3}$	$\mathrm{kg}/m^{3}$
g	Gravitational acceleration	10	$m/s^{2}$
$C_{f}$	Sliding friction coefficient	0.1~0.5	

**Table 2:** Dimensionless parameters.

Parameter	Definition	Expression	Value
$\bar{q}$	Dimensionless heat flux	$\bar{q}=qb/kT_{e}$	0~45
$\bar{h}$	Dimensionless heat transfer coefficient	$\bar{h}=hb/k$	0.1~0.8
*λ*	Radius ratio	λ=a/b	0~1
$\bar{\omega }$	Dimensionless rolling angular velocity	$\bar{\omega }=\omega b^{2}/\psi$	0~10
${\bar{C}}_{T}$	Dimensionless thermal expansion coefficient	${\bar{C}}_{T}=C_{T}T_{e}$	$5\times 10^{-3}$
Ω	Dimensionless parameter	$\Omega =C_{f}\rho gr_{2}/E{\left(\bar{q}{\bar{C}}_{T}sin\;\theta_{0}\right)}^{2}$	0~50

**Table 3:** Energy efficiency improvement of the thick-walled cylindrical rod.

Dimensionless Heat Flux $\bar{q}$	Optimal Radius Ratio *λ*	Energy Efficiency of Solid Rod	Maximum Energy Efficiency	Energy Efficiency Improvement
35	0.31	16.4%	17.51%	1.11%
30	0.49	0%(static)	16.95%	16.95%
20	0.73	0%(static)	16.67%	16.67%

## Supplementary Materials

The following supporting information can be downloaded at: https://www.mdpi.com/article/10.3390/mi13112035/s1, Video S1: Self-sustained rolling of a nylon fiber on a hot surface.

## Appendix A

By substituting Equations (1), (11) and (12) into Equation (13), we have

(A1)
$$M_{z}=\int_{a}^{b}\int_{0}^{2\pi }\left\{\varepsilon -C_{T}\left\{\begin{array}{l} (\pi \bar{h}+\bar{q}\theta_{0})\sum_{m=1}^{\infty }\frac{{\bar{R}}_{0}\left(\lambda ,\bar{h},\bar{r}\right)}{{\bar{F}}_{0}\left(\lambda ,\bar{h}\right)}\\ +2\bar{q}\sum_{n=1}^{\infty }\sum_{m=1}^{\infty }\frac{{\bar{R}}_{n}\left(\lambda ,\bar{h},\bar{r}\right)}{{\bar{F}}_{n}\left(\lambda ,\bar{h}\right)}\frac{\sin\;n\theta_{0}}{n}\frac{\cos\;n\theta +{\bar{\eta }}_{n}\left(\lambda ,\bar{h},\bar{\omega }\right)\sin\;n\theta }{1+\mu_{n}^{2}\left(\lambda ,\bar{h},\bar{\omega }\right)} \end{array}\right\}\right\}r\sin\;\theta rdrd\theta$$

After simplifying Equation (A1), the total bending moment of the rod around Z-axis on the cross section is

(A2) $${\bar{M}}_{z}=2\pi \bar{q}\sin\theta 0{\bar{C}}_{T}\sum_{m=1}^{\infty }\int_{\lambda }^{1}\frac{{\bar{R}}_{1}({\bar{\beta }}_{m},\bar{r})}{{\bar{F}}_{1}({\bar{\beta }}_{m})\left[1+\eta_{1}^{2}({\bar{\beta }}_{m})\right]}{\bar{r}}^{2}d\bar{r}$$

By combining Equations (A2) and (14), the analytical solution of the thermally driven lateral curvature can also be expressed as

(A3) $$\kappa_{z}=\frac{2\pi \bar{q}sin\;\theta_{0}{\bar{C}}_{T}\sum_{m=1}^{\infty }\int_{\lambda }^{1}\frac{{\bar{R}}_{1}({\bar{\beta }}_{m},\bar{r})}{{\bar{F}}_{1}({\bar{\beta }}_{m})\left[1+\eta_{1}^{2}({\bar{\beta }}_{m})\right]}{\bar{r}}^{2}d\bar{r}}{EI}$$

Similarly, by substituting Equations (1), (11) and (16) into Equation (17) and simplifying, the analytical solution for the total bending moment of the rod around x-axis can be obtained as

(A4) $${\bar{M}}_{x}=2\pi \bar{q}sin\;\theta_{0}{\bar{C}}_{T}\sum_{m=1}^{\infty }\int_{\lambda }^{1}\frac{{\bar{R}}_{1}({\bar{\beta }}_{m},\bar{r})}{{\bar{F}}_{1}({\bar{\beta }}_{m})\left[1+\eta_{1}^{2}({\bar{\beta }}_{m})\right]}{\bar{r}}^{2}d\bar{r}$$

Equation (19) can be obtained by substituting Equations (A3) and (A4) into Equation (18) then integrating.
